# Europeans and Malaria

**Published:** 1903-06-27

**Authors:** 


### The Hospital.

A Journal of

### The Medical Sciences and Hospital Administration.

Edited by Sir Henry Burdett, K.C.B.

Vol. XXXIV.—No. 874. [June 27, 1903.

### EUROPEANS AND MALARIA.

The expeditions recently undertaken and still in progress into Nigeria and Somaliland, not to speak of the many other malaria-haunted districts open to British enterprise and promising to reward those who explore them, have added greatly to the practical interest of the recent discoveries concerning the part taken by the mosquito in the diffusion of fevers, and concerning the means by which the dangers hence arising may be obviated. On these questions a very brilliant ray of common sense has recently been shed by Drs. Stephens and Christophers, the Royal Society's Commissioners on Malaria, in a report communicated to the society, and recently published, together with other papers on the same subject, by the Colonial Office, so as to render them accessible to all concerned at the modest price of fourpence-halfpenny. There can be little doubt that the Colonial Office itself, as now organised, will take measures for the distribution of these papers in the localities in which they are chiefly required, but it is none the less desirable that any such measures should be supplemented by private action. Commercial firms with agencies or branches in malarious districts, and the relatives at home of persons who are employed there, should alike make it their business to see that the true principles of prophylaxis, as laid down by Drs. Stephens and Christophers, are made to reach the persons whom they are calculated to protect.

When the prevalence of malarious fever was first traced to the mosquito, the thoughts of those bent upon prevention naturally turned towards the destruction of the insects themselves; and we heard chiefly of measures for filling up the pools in which larvæ were abundant, or for covering their surfaces with a film of petroleum oil, renewed as often as might be required. As the enormous magnitude and costliness of such undertakings became gradually manifest, together with the impossibility of accomplishing them with any approach to completeness, travellers and residents in the localities concerned were advised to put their trust in the careful use of the mosquito net, and in so accommodating their own habits to those of the insects as never to offer to the latter, during their hours of activity, an unprotected surface of skin. There is abundant evidence that, by the use of these precautions, complete immunity from malaria may be secured. But, after all, people living in malarious districts are only human, and only a small proportion of them could be expected to pay the constant attention to matters of detail without which no safety can be attained. The weary traveller would constantly be liable to overlook the existence of a small hole in his mosquito net, or, if he did not overlook it, to trust to the chance that the mosquitoes themselves would do so; and it might often be necessary to finish a journey after sundown, although unprovided with the veils and gloves which prudence would require. On the broad ground of human impatience of detailed precautions, Drs. Stephens and Christophers doubt whether the absolute security which these precautions are calculated to afford will ever be secured in practice; and they have therefore turned their thoughts in other directions. They point out that, after all, the chief source of danger to the European is in the vicinity of native huts; and that a large amount of comparative safety may be secured by simply keeping these huts at a distance.

Recent researches have shown that the blood of a very large percentage of native children contains the malaria parasite in the stage of development in which it is ready to communicate the disease; and the dark and dirty native huts are the favourite haunts and day hiding-places of the anopheles. Whether the children themselves possess some inherited or acquired immunity from the effects of the parasite it would be hard to say, but they constantly carry it in their blood, and their blood forms the natural and staple diet of the mosquitoes belonging to the hut in which they sleep. The mosquitoes, it need hardly be said, are not in themselves dangerous, and only become so after sucking the blood of an infected person. Their flights, according to Drs. Stephens and Christophers, seldom extend to a quarter of a mile, and probably never to half a mile from their places of diurnal shelter. The practical outcome of these considerations is that every native hut should be regarded as a probable source of infection, and that no European should sleep within the danger zone by which each hut is surrounded. In camping or surveying or railway-making the tents of the Europeans should be pitched as far as possible either from a native village or from the tents of the camp followers and their families and, especially when the full distance of half a mile is not attainable, with, if possible, some intervening screen of trees, a hill, or other impediment. The authors point out that this precaution need not in the least interfere with free communication with the natives by day, or with the discharge of their duties as attendants, and that it is no more than the maintenance of the sort of distance which, in India, is always preserved between the native bazaar and the British cantonment.

They mention more than one instance in which the removal of native huts from the compound of a house built for and inhabited by Europeans had entirely removed the liability to fever from which the inmates of the house had never before been free; and they are strongly of opinion that the careful observance of the simple precaution which they indicate would go a very long way towards the removal of the dangers by which residence or travel within malarial zones are at present attended. To live and sleep within the range of the infected mosquito, and to exclude his attentions by nets and gloves, is a matter almost as difficult, and requiring almost as much care, as the preservation of asepticism in surgery. To retire beyond his range, if this be feasible, is within the compass of the ordinary powers of man, and is clearly the course which should now be followed by Europeans.

